# *Mycobacterium hassiacum*: a thermophilic *Mycobacterium* species to demonstrate thermal disinfection of medical devices

**DOI:** 10.1186/s13104-020-04978-7

**Published:** 2020-03-10

**Authors:** Bruno Haas, Kelly J. Soto, Dana S. Day, Alexander C. Roy, Marie-Claude Gagnon, Jodi R. Alt, Philippe Labrie

**Affiliations:** 1 STERIS Canada ULC, 490, Boulevard Armand-Paris, Quebec City, QC G1C 8A3 Canada; 2 STERIS Corporation, Mentor, OH USA

**Keywords:** Medical devices, Thermal disinfection, *Mycobacterium*

## Abstract

**Objective:**

Reprocessing reusable medical devices is crucial in the healthcare industry. To ensure patient safety, strict standards are dictated to validate thermal disinfection in automated washer-disinfectors. The United States Food and Drug Administration (FDA) has specific recommendations on the vegetative bacterial challenge but comparatively vague guidance on the use of a thermophilic *Mycobacterium* strain for thermal disinfection studies. This study aims to compare thermal resistance of *Mycobacterium hassiacum* and *Mycobacterium terrae* and determine which strain is suitable for medical device thermal disinfection validation testing in automated washer-disinfectors.

**Results:**

Thermal resistance was demonstrated in vitro by calculating D-values for each strain at different exposure temperatures, and correlated with actual in situ processing conditions. *M. terrae* was completely killed (> 7 log reduction) at temperatures above 68 °C, with D-values between 46.6 and 27.8 s at temperatures between 59.5 and 67.2 °C. *M. hassiacum* was completely killed (> 8 log reduction) at temperatures above 75 °C, with D-values between 82.1 and 21.7 s at temperatures ranging between 69.2 and 73.6 °C. In vitro results were correlated in a washer-disinfector performance validation setup.

## Introduction

Thousands of reusable medical devices are processed daily at healthcare facilities in order to be re-used on patients. Specially designed washer-disinfectors clean and disinfect reusable medical devices at high throughput following strict standards and guidelines applicable to the field [1, 2]. To ensure patient safety, semi-critical medical devices should ideally be sterilized, or at minimum must be subjected to high-level disinfection [3, 4]. According to the United States Food and Drug Administration (FDA) guidance, thermal disinfection must be demonstrated by a 6-log_10_ reduction of a mixed population of vegetative bacterial strains such as *Pseudomonas aeruginosa*, *Staphylococcus aureus*, *Escherichia coli*, and a representative of the *Klebsiella/Enterobacteria* group (low-level disinfection). In addition, 3- to 6-log_10_ reduction of a thermophilic *Mycobacterium* species must also be achieved for intermediate- and high-level disinfection, respectively [2].

The validation of automated washing and disinfection of reusable medical devices has greatly contributed to the reduction of clinical infections [5]. Chemical disinfection and thermal disinfection are two different processes. Two parameters must be considered to evaluate thermal disinfection: time and temperature of exposure. As per chemical disinfection, the choice of the model organisms is crucial in order to keep a high safety margin. In the late 90s, debates in standards committees raised the need for a surrogate strain representative of *Mycobacterium tuberculosis* and *Mycobacterium avium*-*intracellulare* to safely assess mycobactericidal activity in chemical disinfection of medical devices. In 1998, Griffiths et al. suggested the use of *Mycobacterium terrae* since it possesses similar resistance profile towards disinfectants compared to that of *M. tuberculosis* and *M. avium*-*intracellulare* [6]. However, there are no recommended *Mycobacterium* strains to validate thermal disinfection. The only guidance, from the U.S. FDA, is to use a thermophilic strain [2].

The International Organization for Strandardization’s standard series ISO 15883 addresses thermal disinfection levels using the A_0_ concept that represents a time–temperature correlation of a thermal treatment [7]. It is the equivalent time in seconds at 80 °C delivered by the disinfection process with reference to a microorganism with a *z* value of 10 K [8, 9]. In 2016, McCormick et al. detailed the A_0_ concept and described *Mycobacterium terrae* as a model for thermophilic *Mycobacterium* species [10]. However, no evidence of *M. terrae* thermophilic properties is available. The only reference to *M. terrae* susceptibility to thermal treatment was described in 2011 by Pisot et al. [11]. They found a complete inactivation of *M. terrae* at 65 °C. This behaviour does not correspond with the definition of “thermophilic” [12].

In 1997, Schröder et al. discovered a new *Mycobacterium* species named *Mycobacterium hassiacum* [13]. *M. hassiacum* is characterized as thermophilic as it can show optimal growth at temperatures up to 65 °C [14]. When compared to *M. terrae*, *M. hassiacum* could present substantial benefits when used in a thermal disinfection validation setup. First, it meets the FDA guidance that a thermophilic strain of *Mycobacterium* should be used in thermal disinfection studies. Second, *M. hassiacum* is a fast-growing species; colonies can generally be obtained within 8 days of incubation at 37 °C whereas *M. terrae* is a slow-growing species, with colonies appearing after 14 to 21 days of incubation at 37 °C. Finally, *M. hassiacum* can be easily identified as the test organism, since it has specific phenotypic characteristics including colony shapes and a distinctive yellow color.

In the present study, the thermal resistance of both *M. terrae* and *M. hassiacum* was determined by D-value calculation. The resulting D-values were compared in order to determine which strain could be most suitable as a bacterial challenge in thermal disinfection performance validation of medical washer-disinfectors.

## Main text

### Methods

#### In vitro testing

##### Bacterial seed culture preparation

*Mycobacterium hassiacum* DSM 44199 (Deutsche Sammlung von Mikroorganismen und Zellkulturen, DSMZ, Germany) (equivalent to American Type Culture Collection [ATCC] 700660) [15] and *Mycobacterium terrae* ATCC 15755 (Microbiologics, USA) were grown on Middlebrook 7H11 agar containing 10% oleic acid, dextrose, catalase enrichment (OADC) and incubated at 37 °C for 7 days (*M. hassiacum*) or 14 days (*M. terrae*).

##### Thermal resistance testing

Three independent *M. hassiacum* and *M. terrae* suspensions (at least 10^7^ Colony Forming Units per milliliter [CFU/ml] in 0.85% saline + 0.1% Tween 80) were exposed to thermal treatments of various times and temperatures in a heated water bath. Fractional growth data were used to calculate D-values for each *Mycobacterium* species. A temperature check tube was used to monitor the temperature during treatments.The samples were diluted in peptone water and plated on 7H11 agar supplemented with 10% OADC and incubated at 37 °C for 7 days (*M. hassiacum*) or 14 days (*M. terrae*). Following incubation, CFU were counted to determine bacterial survival using the following formula:

$${\text{T}} = { \log }_{ 10} \left( {{\text{N}}*{\text{D}}} \right)$$

where, T = bacterial titer; N = average number of CFU at the valid dilution; D = dilution factor of the valid dilution.

##### D-value calculation

The D-value is the time required, at a given temperature, to decrease the bacterial population by 1 log_10_.

Treatment temperatures were determined by calculating the average temperature recorded during the treatment time. For each temperature, the average bacterial count (log_10_) was plotted on a graph (Y axis: log_10_ bacterial count; X axis: time of treatment). The D-value for each temperature was calculated using the following formula:

$${\text{D}} = - 1/{\text{slope}}$$

#### Tests in washer-disinfector

##### Bacterial culture and test ampoules

*Mycobacterium hassiacum* ATCC 700660 (Cedarlane, Canada) (equivalent to DSM 44199) [15] was grown at 37 °C in Middlebrook 7H9 broth with glycerol for 7 days. The resulting culture was directly used as test suspension.

*Mycobacterium terrae* ATCC 15755 (Innovation Diagnostics Inc., Canada) was suspended in 0.85% Saline + 0.1% Tween 80 to an optical density at 620 nm wavelength (OD_620_) of 0.1 (bacterial titer estimated > 10^7^ CFU/ml).

1.4 ml of test suspensions of *M. terrae* and *M. hassiacum* (at least 10^7^ CFU/ml) were sealed in glass ampoules and used to test bacterial thermal resistance during a mock thermal phase in a washer disinfector.

##### Washer-disinfector test setup

A single-chamber washer disinfector was connected to a preheated water reservoir. Water at the desired temperature was passed through the washer’s chamber for 1 min on a simulated medical instrument load with ampoules placed at the coldest area as determined per thermal profiling (data not shown). The following five treatment temperatures were tested for a 1-min contact time: 65 °C, 70 °C, 75 °C, 80 °C, and 85 °C. Ampoules of *M. hassiacum* and *M. terrae* were processed at the same time and thus received identical treatments. Once the 1-min water circulation was achieved, the ampoules were placed in ice cold water to immediately stop the heat treatment. The entire content of each ampoule was plated to assess bacterial survival by counting CFUs following 7-days incubation (*M. hassiacum*) or 14-days incubation (*M. terrae*) at 37 °C. The initial titer of each test suspension was also determined to represent the positive control (untreated ampoules).

### Results

#### In vitro testing

**Fig. 1:**
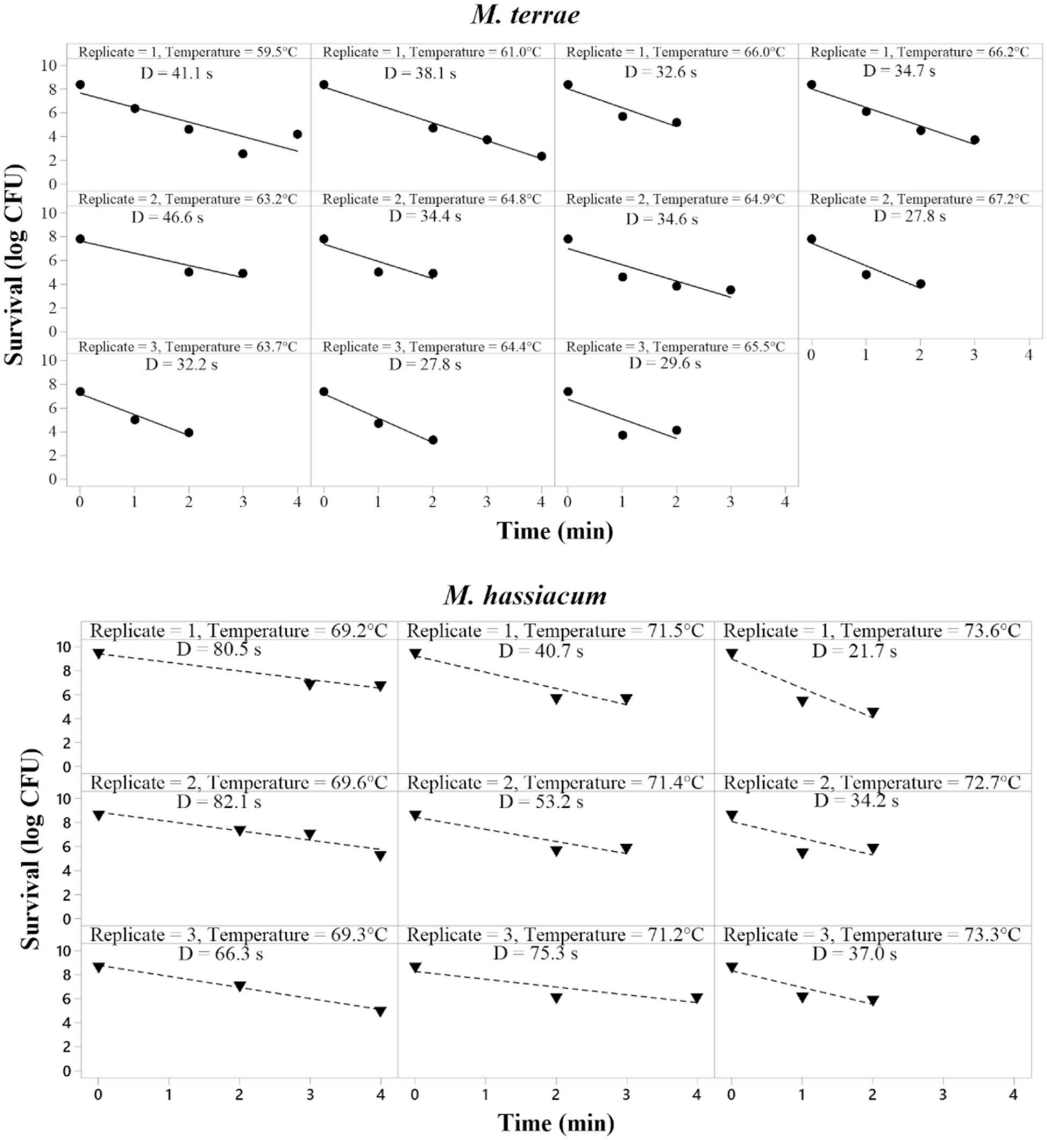
*M. terrae* (upper panel) and *M. hassiacum* (lower panel) survival and calculated D-values as function of treatment time and temperature in vitro

The results of the in vitro thermal treatments including bacterial survival and D-values (in seconds) are shown in Fig. 1.

##### *M. terrae*

Treatments for *M. terrae* were performed at temperatures between 59.5 and 67.2 °C since temperatures higher than 68 °C resulted in complete kill for most of the exposure times tested (data not shown). For all treatment temperatures, bacterial survival of *M. terrae* decreased as treatment time increased (Fig. 1, upper panel). D-values obtained for *M. terrae* ranged between 27.8 and 46.6 s. For replicate 1, D-values of 41.1, 38.1, 32.6, and 34.7 s were obtained for treatments at 59.5, 61.0, 66.0, and 66.2 °C, respectively. Replicate 2 resulted in D-values of 32.2, 27.8, and 29.6 s with treatments at 63.7, 64.4, and 65.5 °C, respectively. D-values obtained on replicate 3 were 46.6, 34.4, 34.6, and 27.8 s for treatments at 63.2, 64.8, 64.9, and 67.2 °C, respectively.

##### *M. hassiacum*

Treatments on *M. hassiacum* suspensions were performed at temperatures between 69.2 and 73.6 °C. Bacterial survival decreased when treatment temperature or time increased (Fig. 1, lower panel). D-values obtained for replicate one were 80.5, 40.7, and 21.7 s for treatments at 69.2, 71.5, and 73.6 °C, respectively. On replicate two, treatments at 69.6, 71.4, and 72.7 °C led to D-values of 82.1, 53.2, and 34.2 s, respectively. The third replicate resulted in D-values of 66.3, 75.3, and 37.0 s at 69.3, 71.2, and 73.3 °C.

Comparison of the highest treatment temperatures for *M. terrae* and the lowest treatment temperatures for *M. hassiacum*, showed that at 67.2 °C, 1-log_10_ reduction of *M. terrae* was achieved after 27.8 s whereas at 69.2 °C, 1-log_10_ reduction of *M. hassiacum* was achieved after 80.5 s.

#### Washer-disinfector testing

**Fig. 2:**
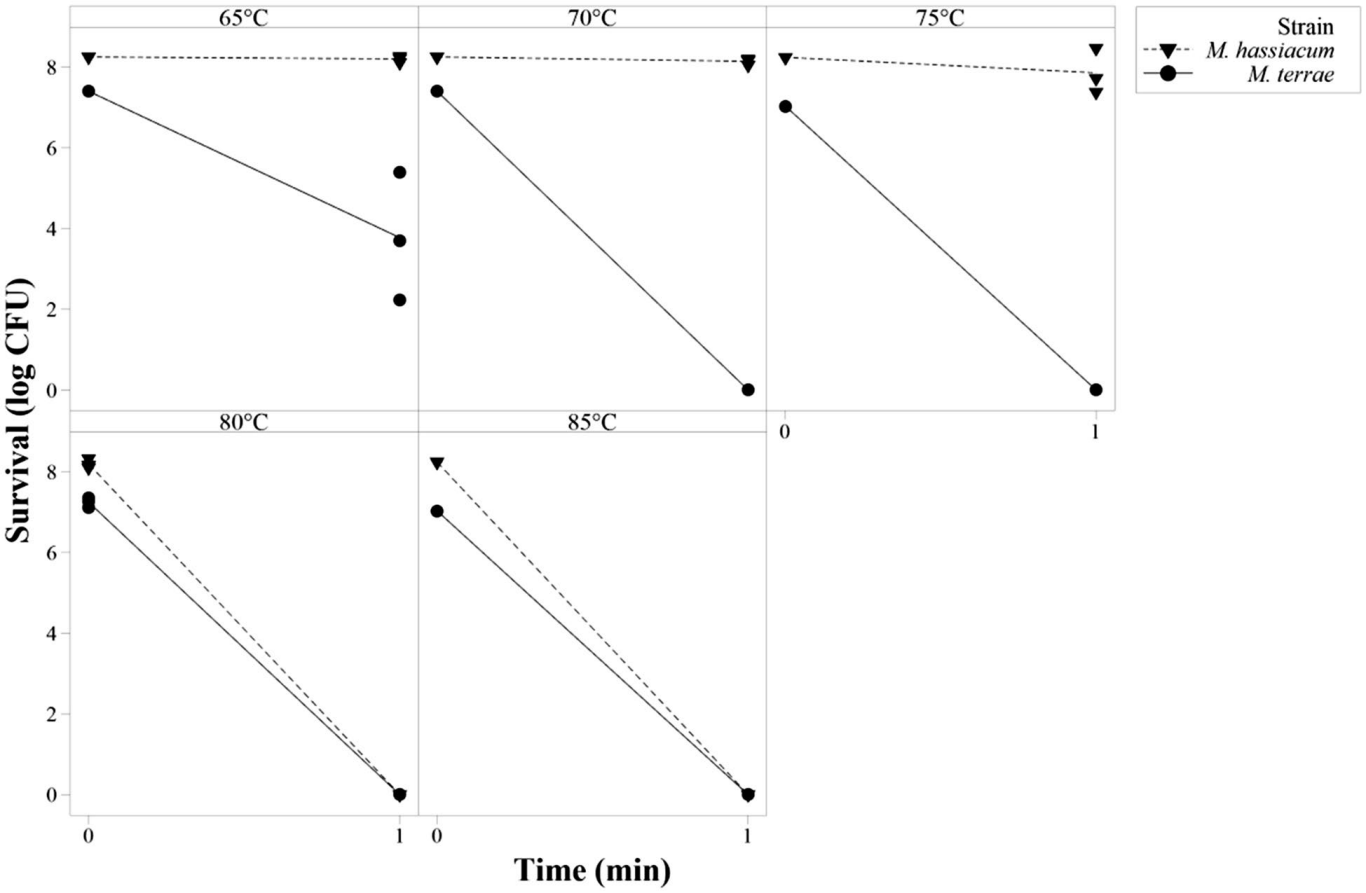
*M terrae* and *M. hassiacum* survival as function of treatment temperature (1 min) in a washer-disinfector

When tested in sealed glass ampoules in a washer-disinfector, it was found that *M. terrae* survival was significantly affected following a 1-min treatment at 65 °C (3.6 log_10_ reduction from 7.4 log_10_ CFU/ml to 3.8 ± 1.6 log_10_ CFU/ml) (Fig. 2). When treatment temperature was increased to 70 °C and above, no surviving CFU were recovered from any ampoules tested for *M. terrae*.

In contrast, no significant reduction of bacterial survival was observed for *M. hassiacum* when treated at 65 °C for 1 min. When temperature was raised to 70 and 75 °C, a 1-min treatment lead to 0.2 and 0.6 log_10_ reduction of the initial titer, respectively. However, the reduction was not significant. When treated for 1 min at 80 and 85 °C, a complete kill of *M. hassiacum* was observed.

### Discussion

*Mycobacterium terrae* has been the surrogate of choice to safely test tuberculocidal activity of chemical disinfectants in the healthcare industry [6]. Another reliable way to disinfect thermo-resistant medical devices is to use an automated washer-disinfector that can perform thermal disinfection. International standard ISO 15883-2 evaluates thermal disinfection in terms of A_0_ values [8, 10, 16]. In addition, bacterial thermal resistance can be expressed using the calculated D-values which represent the time needed to decrease a bacterial population of 1 log_10_ at a given temperature [17].

In vitro testing showed that survival of *M. terrae* was affected beginning at temperature treatments of 60 °C and higher. *M. hassiacum* survival was not affected by treatment temperatures below 68 °C (data not shown). Log_10_ reduction of both *M. terrae* and *M. hassiacum* were time and temperature dependent. However, the two strains were not affected at the same temperature ranges. *M. terrae* depicted a complete kill when submitted to treatments at temperatures higher than 68 °C, which is in accordance with previous observations [11]. Results obtained when testing *M. terrae* in a washer disinfector were in concordance with in vitro data, *i.e.* a complete kill at temperatures above 68 °C.

For critical medical devices such as surgical instruments, the ISO 15883 standard series recommends an A_0_ of 600 for disinfection [1, 7]. This is equivalent to a 1-minute (60 s) treatment at 90 °C. The present study demonstrates that an A_0_ of 60 (1 min at 80 °C) is enough to kill 7 to 8 log_10_ of *M. terrae* or *M. hassiacum* in a laboratory setup. A sufficient safety margin has to be considered for field application; the A_0_ 600 is therefore reasonable to ensure high-level disinfection of critical devices. More importantly, the data collected during this study demonstrate that *M. hassiacum* is more suitable than *M. terrae* as a test organism to validate the tuberculocidal effect of thermal disinfection in an automated washer-disinfector.

To our knowledge, *M. terrae* thermal resistance has never been studied in detail [11], and there is no evidence of its thermophilic behaviour that justifies its use for thermal disinfection validation tests. *M. hassiacum* is a strain that was described for its thermophilic properties [13, 15], and showed in the present study a higher resistance to thermal treatment than *M. terrae*. Therefore, we recommend the use of *M. hassiacum* as the thermophilic *Mycobacterium* species surrogate of choice to validate thermal disinfection in automated washer-disinfectors. The choice of model organisms is crucial to keeping a high safety margin to ensure patient safety when reprocessing medical devices in automated washer-disinfectors.

## Limitations

Variability was observed between replicates. Aggregation of the bacteria could have been causing variable results. *Mycobacteria* are known to aggregate due to their hydrophobicity thus causing difficulties obtaining homogeneous single-cell suspensions.

## Data Availability

Not applicable.

## Abbreviations

U.S. FDA/FDA: United States Food and Drug Administration
ISO: International Organization for Standardization
DSMZ: Deutsche Sammlung von Mikroorganismen und Zellkulturen
ATCC: American Type Culture Collection
OADC: Oleic acid, dextrose, catalase enrichment
CFU: Colonies forming units
OD_620_: Optical density at 620 nm wavelength
